# SG-APSIC1127: Digital transformation of the central sterile supply department: Justify by tracking system

**DOI:** 10.1017/ash.2023.109

**Published:** 2023-03-16

**Authors:** Saharat Kongprajak

**Affiliations:** Central Sterilizing Services Association, Bankok, Thailand

## Abstract

**Background:** Avoidable infections in healthcare (healthcare-associated infections or HAIs) occur globally. The causes of HAI are influenced by a complex combination of gaps in policies, infrastructure, organization, knowledge, healthcare worker behavior, and patient-related factors. Through knowledge, best practices, and infrastructure improvement, the infection prevention and control (IPC) team aims to prevent harm to patients and healthcare workers due to HAIs. The most common HAIs are surgical site infections (SSIs) caused by harmful device-reuse practices, inadequate sterilization, and/or inadequate decontamination procedures. Disinfection and sterilization of instruments and medical devices play very important roles in HAI and SSI prevention. World Health Organization (WHO) Collaborating Centre in Quality Improvement Program certification, the first pilot project in Thailand, included the Central Sterilizing Services Association of Thailand and 15 hospital central sterile supply departments (CSSDs). This quality improvement program for sterilization reprocessing aimed to prevent harm to patients and healthcare workers due to HAIs. **Objectives:** We sought to reduce damage to instruments caused by inadequate reprocessing sterilization to zero incidents. We sought to reduce inadequate packing to ≤3 events per month. We sought to reduce the need to resterilize instruments by >80%. **Methods:** A root-cause analysis meeting was held by CSSD staff, and an IT vendor was consulted about developing an electronic alert system. The following changes were implemented: Staff packed instruments using a list of pictures for each set. Sticker labels were applied showing the proper number of pieces in the set. Identification O-rings were added to instruments with inventory dates, serial numbers, and instructions for use. Stickers were added to indicate the method of sterilization, such as ethylene oxide gas only or hydrogen peroxide only. **Results:** Reports of damage due to the sterilization process decreased to zero. No events related to the packing process were reported, and resterilization of instruments decreased by 98.94%. **Conclusions:** In this project, we implemented a quality improvement process and tracking system, reduced defects, and increased healthcare worker competency to improve patient safety.

